# Galectin-3 as an important prognostic marker for COVID-19 severity

**DOI:** 10.1038/s41598-023-28797-5

**Published:** 2023-01-26

**Authors:** Nevena Gajovic, Sofija Sekulic Markovic, Milena Jurisevic, Marina Jovanovic, Nebojsa Arsenijevic, Zeljko Mijailovic, Marina Jovanovic, Ivan Jovanovic

**Affiliations:** 1 Center for Molecular Medicine and Stem Cell Research, Faculty of Medical Sciences, University of Kragujevac, Kragujevac, Serbia; 2 Department of Infectious Diseases, Faculty of Medical Sciences, University of Kragujevac, Svetozara Markovica 69, 34000 Kragujevac, Serbia; 3 Department of Clinical Pharmacy, Faculty of Medical Sciences, University of Kragujevac, Kragujevac, Serbia; 4 Department of Internal Medicine, Faculty of Medical Sciences, University of Kragujevac, Kragujevac, Serbia; 5 Department of Otorinolaringology, Faculty of Medical Sciences, University of Kragujevac, Kragujevac, Serbia

**Keywords:** Immunology, Immunological disorders, Infectious diseases

## Abstract

Galectin-3 (Gal-3), multifunctional protein plays important roles in inflammatory response, infection and fibrosis. The goal of study was to determine the association of Gal-3, immune response, clinical, biochemical, and radiographic findings with COVID-19 severity. Study included 280 COVID-19 patients classified according to disease severity into mild, moderate, severe and critical group. Cytokines, clinical, biochemical, radiographic data and peripheral blood immune cell make up were analyzed. Patients in critical group had significantly higher serum level of Gal-3, IL-1β, TNF-α, IL-12, IL-10 compared to the patients in less severe stages of disease. Strong positive correlation was detected between Gal-3 and IL-1β, moderate positive correlation between Gal-3, TNF-α and IL-12, moderate negative correlation between Gal-3, IL-10/IL-1β and IL-10/TNF-α. Moderate positive correlation noted between Gal-3 and urea, D dimer, CXR findings. Strong negative correlation detected between Gal-3 and p0_2_, Sa0_2,_ and moderate negative correlation between Gal-3, lymphocyte and monocyte percentage. In the peripheral blood of patients with more severe stages of COVID-19 we detected significantly increased percentages of CD56^−^ CD3^+^TNF-α^+^T cells and CD56^−^ CD3^+^Gal-3^+^T cells and increased expression of CCR5 in PBMCs. Our results predict Gal-3 as an important marker for critical stage of COVID-19. Higher expression of Gal-3, TNF-α and CCR5 on T cells implicate on promoting inflammation and more severe form of disease.

## Introduction

Coronavirus Disease 2019 (COVID-19), highly contagious viral infectious disease is caused by severe acute respiratory syndrome coronavirus 2 (SARS-CoV-2)^1^. In most severe COVID-19 patients, rapid production of pro-inflammatory cytokines, leading to a systemic inflammation and tissue damage in many organs, causing sepsis, acute respiratory distress syndrome (ARDS), respiratory insufficiency, shock or multiple organ failure^2–5^. Elevated plasma levels of Tumor Necrosis Factor alpha (TNF-α), Interleukin (IL)-2, IL-6, IL-10, granulocyte colony stimulating factor (G-CSF), galectins and C-reactive protein (CRP), procalcitonin (PCT), aspartate aminotransferase (AST), alanine aminotransferase (ALT), lactate dehydrogenase (LDH), D dimer, urea, creatinine, Ferritin and lymphopenia in early stage are associated with disease progression to critical illness^3,6^.

Galectin-3, member of the galectin family, is carbohydrate-binding protein located on the cell surface, released mainly by macrophages, endothelial and epithelial cells and plays multiple important roles in virus infections and induce releasing of IL-1β, IL-6, and TNF-α^7–9^. Results in studies by Kazancioglu et al. and Kuśnierz et al. imply increased serum Gal-3 in more severe stages of COVID-19^9,10^.

The aim of study was to analyze the correlation of Gal-3 and other innate immunity cytokines with disease severity.

## Results

### Clinical features of COVID-19 patients

All patients included in study met the criteria for COVID-19^11^ and according to disease severity classified into the four groups. Among them 160 (57.1%) were male. As the disease progresses, statistically significant decrement in percentage of female patients and increment in percentage of male patients was detected (p = 0.041; Table 1). The median age of the patients in the mild group (55.1 ± 1.6) was significantly different from moderate (58.5 ± 1.3), sever (63.5 ± 1.5) or critical one (69.2 ± 1.3) (Table 1). Patients in the moderate, severe and critical groups were more likely to have fever, fatigue, dyspnea, chest pain, auscultatory attenuated breathing sound with audible crackles diffusely or whistling compared with the mild one with statistical significance difference (p < 0.05) (Table 1). The most frequent auscultatory finding in group I was sharpened respiratory sound in 45.7%, normal breathing in 41.4%, attenuated breathing in 40% of patients. Only 8.6% of patients had audible cracks diffusely and 5.7% whistling. Chest X-ray (CXR) findings in group I described like CXR I in 80% and CXR II in 20% of patients, and no one had CXR III, IV, V. In group II, 41.4% of patients had attenuated breathing sound, sharpened respiratory sound 32.9%, audible cracks diffusely 50%, whistling 2.8% of patients. CXR finding are described like CXR II in 60%, CXR III in 40% of patients. In group III the most frequent auscultatory finding was attenuated breathing sound in 44.3% of patients with audible cracks diffusely in 67.1% of patients. All of them had CXR IV. In group IV attenuated breathing sound had 88.6% of patients, audible cracks diffusely 91.4%, whistling 37.1% of patients. All of them had CXR V (Table 1). The length of hospital treatment was longer in the critical group (28 days) compared with the mild group (7 days), moderate (12 days) and severe one (18 days) (Table 1).

**Table 1:** Demographics and clinical characteristics of patients with COVID-19.

	Total (n = 280)	Mild group (n = 70)	Moderate group (n = 70)	Severe group (n = 70)	Critical group (n = 70)	p value*
Age (mean ± SE)		55.1 ± 1.6	58.5 ± 1.3	63.5 ± 1.5	69.2 ± 1.3	*0.001*
Sex						
Female	120 (42.8%)	45 (64.3%)	30 (42.8%)	25 (35.7%)	20 (28.6%)	*0.041*
Male	160 (57.1%)	25 (35.7%)	40 (57.1%)	45 (64.3%)	50 (71.4%)	*0.041*
Clinical manifestations						
Fever	230 (82.1%)	42 (60%)	55 (78.5%)	63 (90%)	70 (100%)	*0.028*
Dry cough	220 (78.6%)	49 (70%)	52 (74.2%)	56 (80%)	63 (90%)	*0.138*
Fatigue	216 (77.1%)	41 (58.6%)	50 (71.4%)	59 (84.2%)	66 (94.2%)	*0.019*
Dyspnea	160(57.1%)	8 (11.4%)	22 (31.4%)	60 (85.76%)	70 (100%)	*0.001*
Myalgia	159 (56.8%)	28 (40%)	36 (51.4%)	45 (64.3%)	50 (71.4%)	*0.017*
Headache	95 (33.9%)	18 (25.7%)	21 (30%)	25 (35.7%)	31 (44.3%)	*0.847*
Nausea and vomitung	91 (31.5%)	36 (51.4%)	27 (38.6%)	17 (24.3%)	11 (15.7%)	*0.117*
Anosmia	82 (29.3%)	28 (40%)	19 (27.1%)	10 (14.3%)	6 (8.6%)	*0.795*
Chest pain	68 (24.3%)	1 (1.4%)	7 (10%)	18 (25.7%)	42 (60%)	*0.004*
Pharyngalgia	28 (13.6%)	13 (18.6%)	11 (15.6%)	9 (12.8%)	5 (7.1%)	*0.124*
Ausculattory findings						
Normal	38 (13.6%)	29 (41.4%)	9 (12.6%)	0 (0%)	0 (0%)	*0.000*
Attenuated breathing sound	150 (53.6%)	28 (40%)	29 (41.4%)	31 (44.3%)	62 (88.6%)	*0.044*
Sharpened respiratory sound	92 (32.9%)	13 (45.7%)	32(32.9%)	39 (41.4%)	8 (11.4%)	*0.206*
Audible cracks diffusely	152 (54.3%)	6 (8.6%)	35(50%)	47 (67.1%)	64 (91.4%)	*0.005*
Audible whistling	90 (32.1%)	4 (5.7%)	17 (2.8%)	27 (21.4%)	42 (37.1%)	*0.067*
CXR findings						
Normal finding	56 (20%)	56 (80%)	0 (0%)	0 (0%)	0 (0%)	*0.001*
Interstitial thickening	56 (20%)	14 (20%)	42 (60%)	0 (0%)	0 (0%)	*0.001*
Focal consolidation	28 (10%)	0 (0%)	28 (40%)	0 (0%)	0 (0%)	*0.001*
Multifocal consolidation	70 (25%)	0 (0%)	0 (0%)	70 (100%)	0 (0%)	*0.001*
Diffuse alveolar changes—ARDS	70 (25%)	0 (0%)	0 (0%)	0 (0%)	70 (100%)	*0.001*
Length of hospital stay (in days)		7	12	18	28	*0.154*

Significant values are in italics.

Data expressed as mean, standard error (SR), frequency (percentage).

*p values indicate differences between mild, moderate and severe group p < 0.05 was considered statistically significant.

Significant differences in laboratory features noted between defined group. Patients in moderate, severe and critical group had an increase with statistically significant differences (p < 0.05) in white blood cell count, neutrophil count, levels of glycemia, urea, creatinine, TBIL, DBIL, AST, ALT, LDH, CK, D-dimer, CRP, PCT, Fe, Ferritin and reduced lymphocyte count, monocyte count, decreased levels of albumin, lower values of Sa0_2_ and p0_2_ (Table 2). There were no statistically significant differences in number of erythrocytes and platelets, levels of hemoglobin, K^+^, Na^+^, Fe, pH of blood, pC0_2_ (Table 2).

**Table 2:** Laboratory findings of patients with COVID-19.

Measured parameters	Normal range	Mild group (n = 70)	Moderate group (n = 70)	Severe group (n = 70)	Critical group (n = 70)	p value*
White blood cell count, × 10^9^/L	3.7–10.0	6.8	8.4	9.9	10.1	*0.001*
Neutrophil count %	44.0–72.0	69.8	76.4	79.3	85.0	*0.001*
Lymphocyte count %	20.0–46.0	19.0	16.0	13.1	9.7	*0.001*
Monocyte count %	2.0–12.0	8.9	7.8	6.1	5.5	*0.001*
Eritrocite count 10^12^/l	4.34–5.72	4.4	4.4	4.6	4.6	*> 0.05*
Trombocite count 10^9^/l	135–450	227	239	247	265	>* 0.05*
Hemoglobin g/L	138–175	135	129	128	127	*> 0.05*
Glucose mmol/L	3.8–6.1	6.7	7.2	8.6	9.6	*0.003*
Urea mmol/L	3.0–8.0	6.6	7.7	8.1	10.9	*0.001*
Creatinin umol/L	49–106	97.5	98.6	99.1	142.2	*0.001*
BILT umol/L	0.0–21.0	10.9	12.8	13.7	14.4	*0.027*
BILD umol/L	0.0–6.6	2.7	3.2	3.7	4.2	*0.019*
AST U/L	0–40	39.3	54.1	59.9	66.6	*0.001*
ALT U/L	0–40	49.1	79.7	99.4	126.8	*0.001*
Albumin g/L	35–52	37.6	35.6	33.8	32.3	*0.001*
LDH U/L	220–450	517.4	629.8	860.5	919.8	*0.001*
CK U/L	0–171	157.1	195.7	277.0	399.4	*0.001*
D dimer ug/ml	< 0.50	1.5	2.3	3.5	4.6	*0.001*
CRP mg/L	0.0–5.0	59.3	114.1	140.4	128.7	*0.001*
PCT ng/mL	0.5–2.0	0.1	0.2	0.5	1.7	*0.001*
pO_2_ kPa	10.7–13.3	10.5	7.9	7.1	5.2	*0.001*
pCO_2_ kPa	4.7–6.0	4.5	4.8	4.6	4.9	> *0.05*
SaO2%	95–98	94.4	89	82.6	74	*0.001*
ph	7.35–7.45	7.5	7.3	7.4	7.5	>* 0.05*
K + mmol/L	3.5–4.5	3.7	3.6	3.3	3.1	> *0.05*
Na + mmol/L	136–145	136.8	137.9	133.8	135.9	> *0.05*
Fe umol/L	6.6–26	6.5	6.2	5.8	4.9	> *0.05*
Feritin ug/L	20–300	521.3	884.9	1230.6	1699.1	*0.001*

Significant values are in italics.

Data expressed as median.

*p values indicate differences between mild, moderate and severe group p < 0.05 was considered statistically significant.

### Patients with severe COVID-19 had increased Gal-3, IL-10 and proinflammatory cytokines

**Figure 1:**
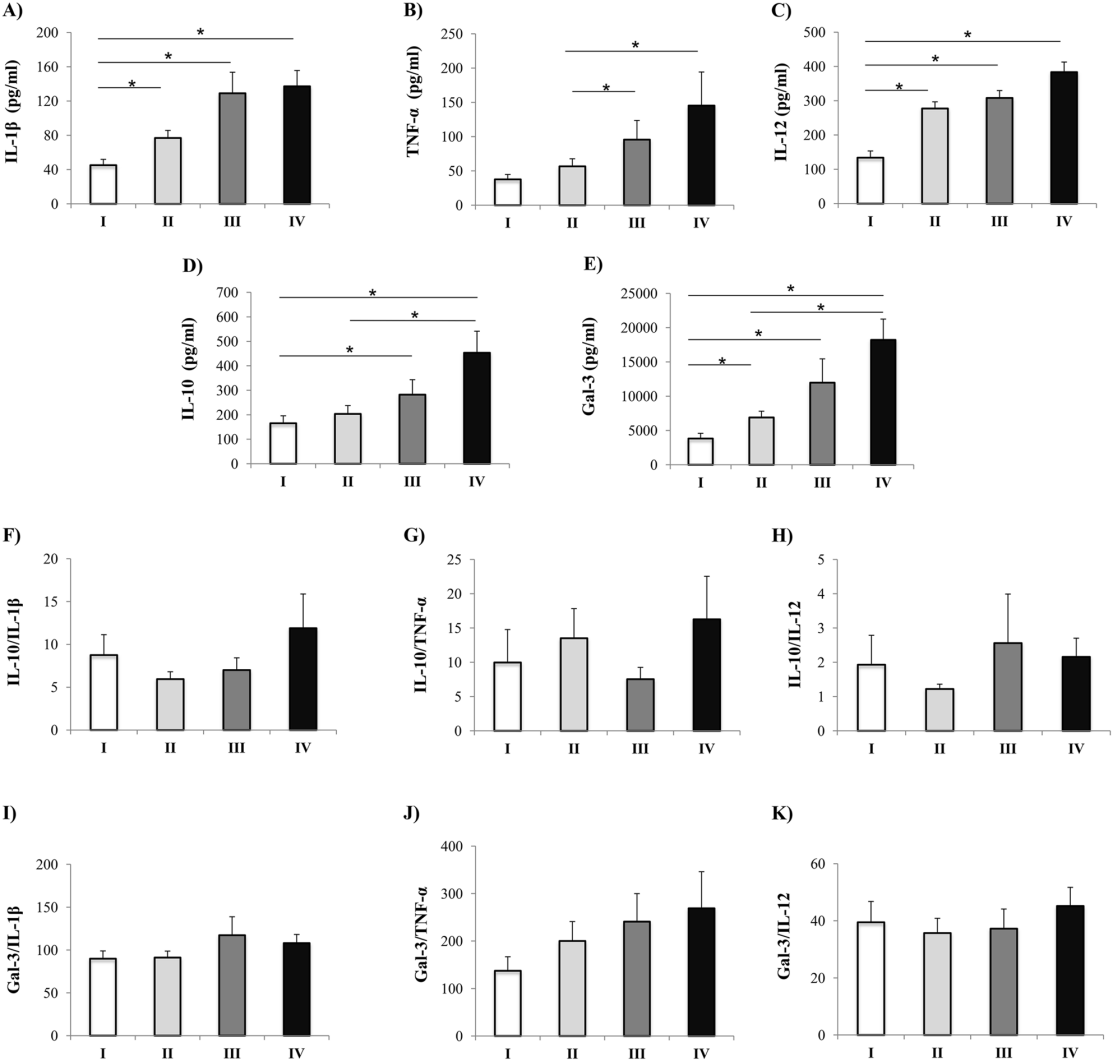
Systemic values and ratios of innate immunity cytokines. According to the disease severity, all Coronavirus disease 2019 (COVID-19) patients were divided in four groups (stage I (n = 61), II (n = 69), III (n = 54), IV (n = 48)). Systemic values of tumor necrosis factor α (TNF-α), interleukin (IL) 1β, IL-12, IL-10, and Galectin-3 were determined by ELISA. The ratios of IL-10/IL-1β, IL-10/TNF-α, IL-10/IL-12 as well as Gal-3/IL-1β, Gal-3/TNF-α and Gal-3/IL-12 were evaluated for each patient, separately. For statistical significance determination, Kruskal–Wallis test, with post hoc Mann–Whitney Rank Sum test, was used. *p < 0.05.

Analyses of cytokines revealed that patients in stage II had significantly higher level of IL-1β (p = 0.004) and IL-12 (p = 0.001) in comparison to stage I. Similarly, stage III patients had increased concentration of IL-1β (p = 0.001), IL-12 (p = 0.001) and IL-10 (p = 0.009) compared to patients in stage I and TNF-α (p = 0.012) compared to patients in stage II. Patients in stage IV of COVID-19 had significantly higher serum level of pro-inflammatory cytokines IL-1β (p = 0.001), TNF-α (p = 0.012) and IL-12 (p = 0.001) as well as immunosuppressive IL-10 (p = 0.001) compared to the patients in less severe stages of disease (Fig. 1A–D). Analyses of Gal-3 serum concentration revealed its increment as the disease progresses, precisely in stage II (p = 0.014), stage III (p = 0.001) and stage IV (p = 0.001) compared to stage I patients (Fig. 1E). Ratios of serum levels of IL-10 and Gal-3 with IL-1β, TNF-α and IL-12 in all stages of COVID-19 were made. No differences were obtained in ratios IL-10/IL-1β, IL-10/TNF-α, IL-10/IL-12 as well as Gal-3/IL-1β, Gal-3/TNF-α, Gal-3/IL-12 between defined groups (Fig. 1F–K).

### Increased expression of Gal-3, TNF-α and CCR5 in PBMCs in patients with severe COVID-19

**Figure 2:**
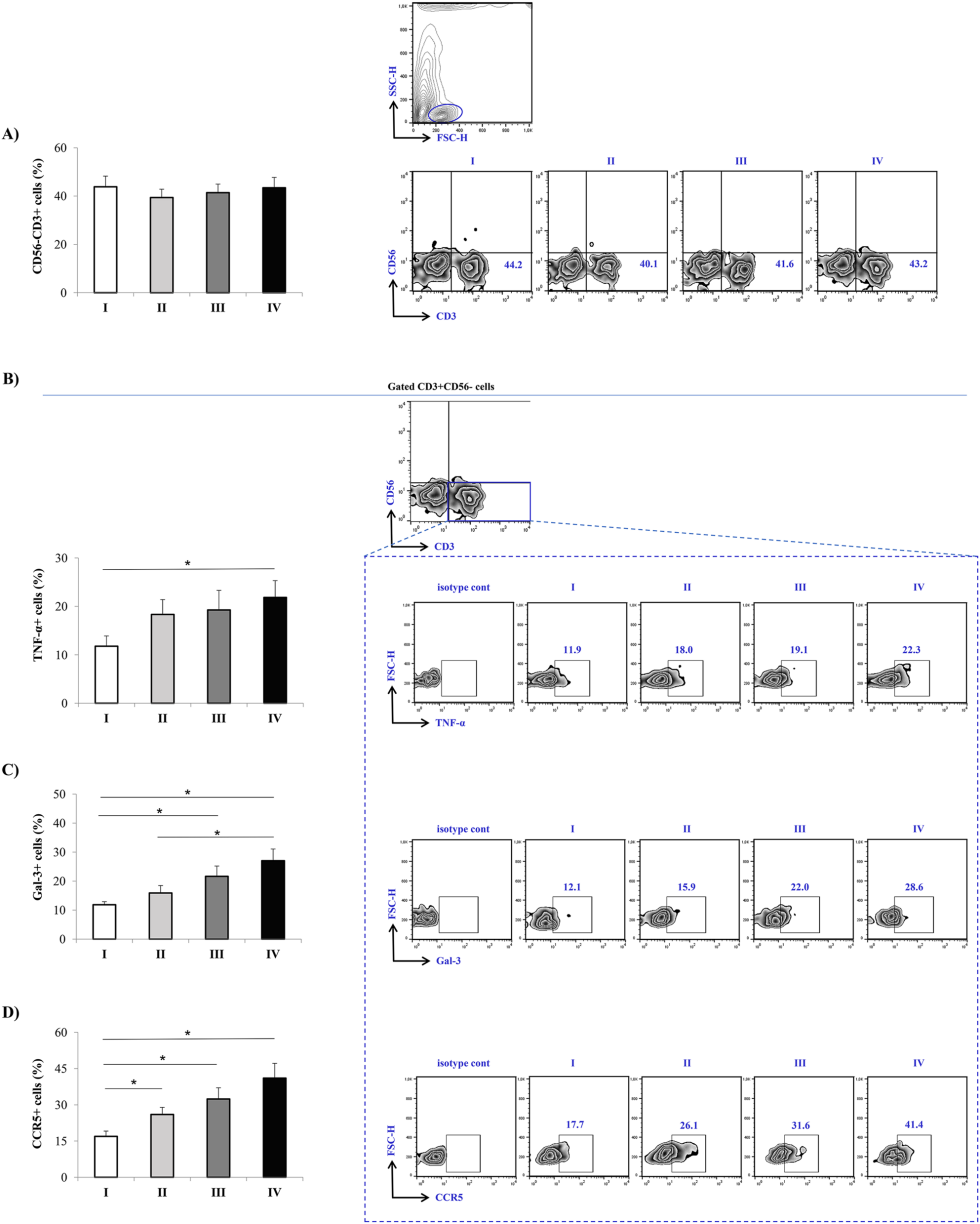
Increment in percentage of TNF-α^+^, Gal-3^+^, and CCR5^+^ T cells in stage IV patients with COVID-19. The graph and representative FACS plots displaying the percentage of CD56^-^CD3^+^, CD56^−^CD3^+^ TNF-α^+^ cells, CD56^−^CD3^+^ Gal-3^+^ cells, CD56^−^CD3^+^ CCR5^+^ cells, among PBMCs of patients in all progressive stages of COVID-19 (stage I (n = 19), II (n = 21), III (n = 22), IV (n = 24). Isotype control for each measured marker is also displayed. The Kruskal–Walli’s test (with post-hoc Mann–Whitney *U*-test) was applied to evaluate statistically significant differences. *p < 0.05.

**Figure 3:**
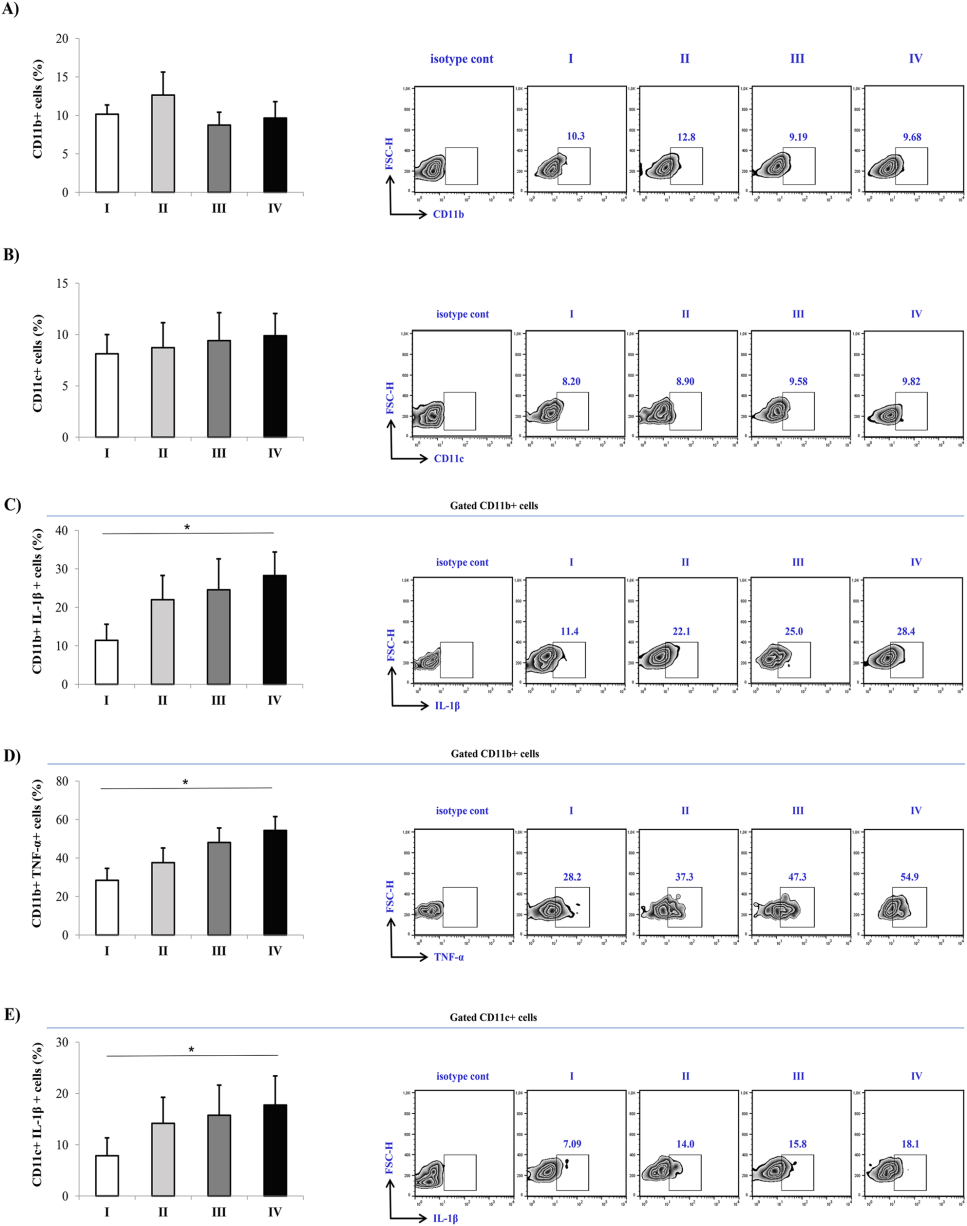
Increment in percentage of TNF-α^+^, and IL-1β ^+^ dendritic cells and monocytes in stage IV patients with COVID-19. The graph and representative FACS plots displaying the percentage of CD11b^+^, CD11b^+^ IL-1β ^+^, CD11b^+^ TNF-α^+^ cells, CD11c^+^, CD11c^+^ TNF-α^+^ cells, among PBMCs of patients in all progressive stages of COVID-19 [stage I (n = 19), II (n = 21), III (n = 22), IV (n = 24)]. Isotype control for each measured marker is also displayed. The Kruskal–Walli’s test (with post-hoc Mann–Whitney *U*-test) was applied to evaluate statistically significant differences. *p < 0.05.

Flow cytometry analysis of lymphocytes in peripheral blood did not detect any statistically significant difference in the percentage of CD56^−^CD3^+^T cells between defined groups (Fig. 2A). Significantly increased percentages of CD56^−^CD3^+^TNF-α^+^T cells (p = 0.050) as well as CD56^−^CD3^+^ Gal-3^+^T cells (p = 0.007) were detected in the peripheral blood of patients in stage IV compared to the patients in other stages (Fig. 2B,C). Patients in more severe stages of the disease showed increased expressions of CCR5 in T cells as observed by higher percentage of CD56^−^CD3^+^CCR5^+^T cells in stages II (p = 0.035), III (p = 0.023) and mainly stage IV (p = 0.005) when compared to stage I patients (Fig. 2D). Analyses of dendritic cells (DCs) and monocytes revealed that there were no differences in the percentage of CD11b^+^ cells or CD11c^+^ cells in peripheral blood derived from patients in different stages of COVID-19 (Fig. 3A,B). Higher percentage of CD11c^+^IL1-β^+^ (p = 0.078), and significantly higher percentage CD11b^+^IL1-β^+^ (p = 0.006) and CD11b^+^TNF-α^+^ (p = 0.012) cells were measured in stage IV (Fig. 3C–E).

### Strong correlation between Gal-3 and proinflammatory cytokines

Next, analyses of correlation between Gal-3 and pro- and anti-inflammatory cytokines and their ratios in COVID-19 patients were performed. Strong positive correlation was detected between Gal-3 and IL-1β (p = 0.001), moderate positive correlation between Gal-3 and TNF-α (p = 0.001) and IL-12 (p = 0.001) (Table 3). Moderate negative correlation noted between Gal-3, IL-10/IL-1β (p = 0.001) and IL-10/TNF-α (p = 0.001) (Table 3).

**Table 3:** Correlation between Gal-3 and pro- and anti-inflammatory cytokines.

	Gal-3	
	Pearson’s rho	p value*
IL-1β	**0.723**	*0.001*
TNF-α	**0.228**	*0.001*
IL-12	**0.437**	*0.001*
IL-10	0.062	*0.348*
IL-10/IL-1β	**− 0.408**	*0.001*
IL-10/TNF-α	**− 0.355**	*0.001*

Significant values are in bold and italics.

*p value < 0.05 was considered statistically significant.

### Moderate correlation between Gal-3 and clinical parameters of COVID-19

Further, correlation between Gal-3 and clinical parameters of COVID-19 has been done. Results revealed moderate positive correlation between Gal-3 and D dimer (p = 0.001), CXR findings (p = 0.001) and urea (p = 0.001), weak positive correlation between Gal-3 and PCT (p = 0.001), glycemia (p = 0.004), neutrophils no. (p = 0.001), CRP (p = 0.011), LDH (p = 0.002), AST (p = 0.020) and creatinine (p = 0.028) (Table 4). Weak negative correlation was detected between Gal-3 and monocytes no. (p = 0.003), while moderate negative correlation was measured between Gal-3 and p0_2_ (p = 0.001), Sa0_2_ (p = 0.001) and lymphocyte percentage (p = 0.001) (Table 4).

**Table 4:** Correlation between Gal-3 and laboratory findings.

	Gal-3	
	Pearson’s rho/Spearman's rho	p value*
pO_2_	**− 0.320**	*0.001*
PCT	**0.257**	*0.001*
D-dimer	**0.300**	*0.001*
CXR	**0.340**	*0.001*
Neutrophil count	**0.213**	*0.001*
Lymphocyte count	**− 0.249**	*0.001*
Monocyte count	**− 0.195**	*0.003*
Eritrocite count	**− 0.135**	*0.040*
Glucose	**0.190**	*0.004*
Urea	**0.339**	*0.001*
Creatinine	**0.145**	*0.028*
AST	**0.156**	*0.001*
Albumin	**− 0.234**	*0.001*
LDH	**0.219**	*0.002*
CRP	**0.166**	*0.011*
SaO_2_	**− 0.300**	*0.001*
Na^+^	**0.130**	*0.049*

Significant values are in bold and italics.

*p value < 0.05 was considered statistically significant.

The univariate logistic regression analysis showed that age (> 65 years), dyspnea, chest pain, attenuated breathing sound, audible cracks diffusely, platelet count, lymphocyte, albumin, urea, creatinin, d-dimer, CRP, PCT, IL-1β (> 120 pg/mL), IL-10 (> 300 pg/mL), TNF-α (> 65 pg/mL) and Gal-3 (> 5000 pg/mL) were associated with critical stage of COVID-19 (Table 5). Multivariate logistic regression analysis identify that Gal-3 (OR 2.97, 95% CI 1.006–8.764; p = 0.049), IL-10 (OR 3.117, 95% CI 1.119–8.105; p = 0.020), creatinine (> 100 umol/L) (OR 4.035, 95% CI 1.549–10.506; p = 0.004), platelet count (< 100 × 10^9^/L) (OR 9.608, 95% CI 1.255–73.535; p = 0.029), chest pain (OR 4.964, 95% CI 1.158–21.278; p = 0.031), attenuated breathing sound (OR 4.703, 95% CI 1.601–13.812; p = 0.005) and audible cracks diffusely (OR 2.467, 95% CI 1.006–6.048; p = 0.048) were independently significant factors for critical stage of COVID-19.

**Table 5:** Factors associated with critical stage of COVID-19.

	Univariate logistic regression			Multivariate logistic regression		
	OR	95% CI	*p* value*	OR	95% CI	*p* value*
Age (> 65 years)	3.513	1.891–6.525	*0.001*	*–*	*–*	*–*
Males	1.187	0.606–2.324	*0.617*	*–*	*–*	*–*
Dyspnea	2.608	1.451–4.690	*0.001*	*–*	*–*	*–*
Chest pain	2.613	1.108–6.165	*0.028*	4.964	1.158–21.278	*0.031*
Attenuated breathing sound	1.925	1.048–3.534	*0.035*	4.703	1.601–13.812	*0.005*
Audible cracks diffusely	1.829	1.013–3.302	*0.045*	2.467	1.006–6.048	*0.048*
Platelet count (< 100 × 10^9^/L)	6.338	1.991–20.179	*0.002*	9.608	1.255–73.535	*0.029*
White blood cell (> 10 × 10^9^/L)	1.834	0.966–3.481	*0.064*	*–*	*–*	*–*
Lymphocyte (< 0.6 × 10^9^/L)	3.387	1.762–6.570	*0.001*	*–*	*–*	*–*
Albumin (< 30 g/L)	2.112	1.103–4.045	*0.024*	*–*	*–*	*–*
Urea (> 6 mmol/L)	3.815	1.918–7.589	*0.001*	*–*	*–*	*–*
Creatinine (> 100 µmol/L)	4.103	2.251–7.476	*0.001*	4.035	1.549–10.506	*0.004*
D dimer (> 1.1 µg/mL)	3.920	2.078–7.392	*0.001*	*–*	*–*	*–*
CRP (> 100 mg/L)	1.811	1.013–3.237	*0.046*	*–*	*–*	*–*
PCT (> 1 ng/mL)	4.409	2.049–9.448	*0.001*	*–*	*–*	*–*
IL-1β (> 120 pg/mL)	2.342	1.201–4.567	*0.012*	*–*	*–*	*–*
IL-10 (> 300 pg/mL)	2.630	1.351–5.119	*0.004*	3.117	1.119–8.105	*0.020*
IL-12 (> 350 pg/mL)	1.910	0.981–3.720	*0.057*	*–*	*–*	*–*
TNF-α (> 65 pg/mL)	2.029	1.030–3.997	*0.041*	*–*	*–*	*–*
Gal-3 (> 5000 pg/mL)	2.467	1.294–4.714	*0.006*	2.97	1.006–8.764	*0.049*

Significant values are in italics.

*p value < 0.05 was considered statistically significant.

## Discussion

In this study, we analyzed the relationship between Gal-3 and innate immunity cytokine profile (TNF-α, IL-1β, IL-10, IL-12), functional phenotype of lymphocytes, monocytes and dendritic cells, clinical, biochemical, and radiographic outcomes with disease severity. Patients with COVID-19 were classified into the mild, moderate, severe and critical group. We found correlation between gender and age with disease severity. Most patients in critical group were male (71.4%) and older (over 68 years), meaning older male patients are more prone to develop the most severe stage of COVID-19 with worse clinical symptoms. Our results are in line with the studies of Statsenko et al., Liu et al. and Meng et al. where authors suggested that age and gender corelates with disease severity confirming that increased risk of developing the most severe form of COVID-19 is often in elderly and male patients^12–14^. Frequency of fever, fatigue, dyspnea, chest pain, auscultatory attenuated breathing sound, crackles, whistling was significantly higher in moderate, severe and critical groups compared with the mild one (Table 1).

As disease progresses, we noted increased values of white blood cell count, neutrophil count, values of urea, glycemia, creatinine, BILD, BILT, AST, ALT, CK, LDH, D-dimer, CRP, PCT, Ferritin, as well as decreased values of lymphocyte and monocyte count, albumin, Sa0_2_ and p0_2_. The same findings were reported in studies of Rathores et al. and Bairwa^15,16^.

COVID-19 can affect lungs, heart, kidneys, gastrointestinal tract, and brain by specific host defense responses associated with inflammatory activity and coagulopathy^2,17,18^ . In patients with severe and critical features occurs uncontrolled immune-inflammatory response with rapid production of cytokines of IL-1 family, TNFα, IL-6, granulocyte-colony stimulating factor and several chemokines^19^. Significantly higher sera levels of PCT were noted in severe and critical group and might be marker of cytokine storm or multiple infections^20^. As the clinical picture of the patients deteriorated, the damage to the lung tissue was bigger. These results suggest a correlation between CXR findings and disease severity and indicate significant difference between CXR findings in defined groups.

We measured serum values of proinflammatory cytokines IL-1β, TNF-α and IL-12 and anti-inflammatory IL-10. During COVID-19 progression, abnormal levels of IL-1β, TNF-α, IL-2, IL-7, IL-12, macrophage colony-stimulating factor (M-CSF), granulocyte colony-stimulating factor (G-CSF), and others can be detected in patient’s blood^21,22^. IL-10 is anti-inflammatory cytokine important for immune response suppression and tissue damage restriction. Several studies confirmed dramatically increment of IL-10 in COVID-19 patients^23,24^. Possible explanation is that parallel with rising of proinflammatory cytokines, IL-10 increases in order to limit inflammation^25^. Our results showed significantly higher level of IL-1β, TNF-α, IL-12 and IL-10 in patients with stage IV of COVID-19 in comparison to milder forms of the disease (Fig. 1). These results are in line with previous studies confirming growing level of inflammation as COVID-19 progresses. The almost unchanged ratio between IL-10 and proinflammatory cytokines during COVID-19 progression (Fig. 1) points on similar dynamics of all cytokine’s growth.

Flow cytometry analyses revealed higher percentages of TNF-α^+^T cells, IL1-β producing dendritic cells and IL1-β^+^ and TNF-α producing monocytes in the peripheral blood of patients in the stage IV. (Fig. 2). Previous study confirmed that cytokine release syndrome is in positive correlation with the degree of COVID-19 severity, which is depicted by higher production of proinflammatory cytokines^26^. It has been shown that TNF-α can directly propagate production of other proinflammatory cytokines such as IL-6 and IL-1β^27^. In line with these confirmations is our result showing predomination of TNF-α producing T cells, IL1-β producing dendritic cells and IL1-β^+^ and TNF-α producing monocytes in the most severe stage of the disease (Fig. 2). Higher numbers of TNF-α/IL1-β producing T cells/dendritic cells/ monocytes represent most likely source of increased systemic TNF-α and IL1-β. CCR5 is a protein expressed constitutively on many immune and non-immune cells involved in different immune processes. Our analyses showed significantly higher expression of CCR5 on T cells in stage IV compared to milder forms of COVID-19 (Fig. 2). During COVID-19, infected airway epithelial cells increase production of CCL5 that functions as chemotactic molecule by binding to CCR5^28^. So, higher expression of CCR5 on T cells can enable linking of CCL5 to CCR5 stimulating migration of T lymphocytes in patient’s lungs and promoting inflammation and more severe form of disease. This result explains reduced lymphocyte count in patients with more severe COVID-19 (Table 2).

As different studies confirmed that Gal-3 can act as stimulative or inhibiting molecule, the next goal of our study was analysis of Gal-3 in COVID-19 patients^29,30^. Significantly higher level of Gal-3 was detected in sera of patients in stage IV in comparison to patients in other stages of disease (Fig. 1). This result is in line with studies of Kazancioglu et al. and Cervantes-Alvarez et al. showing higher levels of Gal-3 in the patients with severe COVID-19^10,31^. We further investigate Gal-3 expression in T cells from peripheral blood. Flow cytometry analyses showed that patients in stage IV of COVID-19 had significantly higher percentage of Gal-3^+^ T cells compared to patients with milder disease (Fig. 2). Increased production of Gal-3 in T cells may be the source of elevated systemic Gal-3 in patients with severe form of COVID-19. Previous study showed that Gal-3 placed in the serum or on the cell membrane can increase inflammation via stimulation of migration and infiltration of neutrophils and other proinflammatory cells and massive production of different proinflammatory cytokines to the infected site^32,33^. It is possible that after migration to lung via CCR5-CCL5 interaction, T cells by expressing Gal-3 amplify airway inflammation via attracting immune cells and stimulating production of proinflammatory cytokines. After being released from the cell, Gal-3 can link to receptors on innate immune cells and act as alarmin by stimulating production of TNF-α, IL-1β, IL-6, IL-12^34^. These potential actions of Gal-3 are substantiated by increased systemic values of TNF-α, IL-1β, and IL-12 (Fig. 1) as well as strong positive correlation that is measured between Gal-3 and IL-1β and moderate positive correlation between Gal-3 and TNF-α and IL-12 (Table 3). As part of innate immunity, inflammasomes are receptors and sensors that can activate caspase-1 and facilitate inflammation in response to microorganisms^35^. Recent study showed that during COVID-19, as a response to the presence of Corona virus, human macrophages induce inflammasomes activity, that is followed by secretion of IL-1β and IL-18 and the extension of inflammation in lungs^36^. Moreover, some studies explained that in different diseases Gal3 can stimulate the function of inflammasome thus inducing proinflammatory process^37^. According to these data, it is possible that besides direct effect of Corona virus on the function of inflammasome, indirectly gal-3 can also potenatiate inflammation via inflammasome activity. Interestingly, elevated systemic values of Gal-3 were detected in patients in stage IV of COVID-19. This group is dominated by older male patients. As it is already known that Gal-3 levels in sera increase with age and have been associated with different diseases very frequent in the elderly population such as cardiovascular disease^38^, it appears that elevated Gal-3 may be due to aging itself.

Gal-3 significantly correlated with several biomarkers and clinical parameters (Table 4). Moderate positive correlation detected between Gal-3 and D dimer, CXR findings and urea. Moderate negative correlation noted between Gal-3 and p0_2_, Sa0_2_, lymphocyte and monocyte percentage (Table 4). All these biomarkers and parameters important for monitoring of COVID-19 patients correlate with Gal-3 and point on potentially important pathophysiological role of Gal-3 in COVID-19.

Our results revealed that Gal-3 could predict critical stage of COVID-19. According to our findings, systemic Gal-3 could be a valuable marker for COVID-19 severity.

We found higher systemic values of Gal-3, IL-10 and proinflammatory cytokines in patients with critically COVID-19. The increment of systemic Gal-3 is followed by increased expression of Gal-3 and chemokine CCR5 in T cells, increased production of TNF-α and IL1-β from PBMCs. Systemic values of Gal-3 strongly correlate with proinflammatory cytokines and clinical parameters of disease severity.

Taking all these in account we believe that Gal-3 may facilitate acquired proinflammatory immune response, and with intense innate pro-inflammatory immune response leads to severe inflammation in the lungs and poor outcome, which makes it a promising therapeutic target.

## Methods

### Ethical statement

This study was performed in the University Clinical Center of Kragujevac (Covid Center) and Faculty of Medical Sciences (Center for Molecular Medicine and Stem Cell Research), University of Kragujevac, Serbia. All examined subjects gave informed consent. Ethics Committees of the University Clinical Center of Kragujevac (Approval Number 01/20-406) and Faculty of Medical Sciences, University of Kragujevac (Approval Number 01-6776) gave ethical approvals. All research procedures were carried out in accordance with the Declaration of Helsinki and the Principle of Good Clinical Practice.

### Participants

In this observational and cross-sectional study 280 patients with COVID-19 were included and divided into four groups: mild, moderate, severe, critical group.

1. Mild group consists of 70 patients with: fever (37–38 °C), fatigue, myalgia, nausea, vomiting, headache, anosmia, pharyngalgia, discreet dry irritating cough, with Sa0_2_ 92%-100%, p0_2_ 8.5–13.3 kPa, with normal or attenuated respiratory noise, with normal or interstitial thickening in CXR findings (CRX I, II).
2. Moderate group consists of 70 patients with fever (38–39 °C), dry irritating cough, fatigue, dyspnea, myalgia, nausea, vomiting, headache with Sa0_2_ 83–91%, p0_2_ 7.2–8.4 kPa with attenuated/sharpened respiratory noise, audible cracks in the lower segments of the lungs, with interstitial thickening or focal zones of consolidation in CXR findings (CRX II, III).
3. Severe group consists of 70 patients with fever (39–39.5 °C), frequent dry irritating cough, dyspnea, fatigue, chest pain, with Sa0_2_ 75–82%, p0_2_ 5.6–7.1 kPa which require high frequency ventilation (HFV), non-invasive mechanical ventilation (Non Invasive Ventilation, NIV), with auscultatory attenuated/inaudible respiratory noise, audible whistling/crackles diffusely, with multifocal zones of consolidation in CXR lung findings (CRX IV);
4. Critical group consists of 70 patients with fever over 39.5–40 °C, dyspnea, frequent dry irritating cough, fatigue, chest pain, Sa0_2_ ≤ 68–74%, p02 ≤ 4.2–5.5 kPa which require invasive mechanical ventilation, with auscultatory attenuated respiratory noise, audible crackles/whistling diffusely and ARDS in CXR lung findings (CRX V).

To reduce the risk of bias, all physical examinations of the patients were performed by two specialists in internal medicine and infectious diseases, independently of each other, while CXR lung findings were evaluated as previously described^34^ by a radiology specialist who was not involved in the treatment of the patients. Patients with inconsistencies in breath sounds with chest X-ray findings were excluded from the study.

### Data collection

Blood samples were drawn by venous puncture from all patients, for complete blood count, D dimer, urea, glycemia, creatinine, CRP, PCT, creatinine kinase (CK), AST, ALT, LDH, albumin (ALB), total bilirubin (TBIL), direct bilirubin (DBIL), Fe, Ferritin, K^+^, Na^+^. To determine the serum concentration of cytokines and glycan—binding molecules, 10 mL of blood was taken from all patients by venipuncture. As described in previous study, the blood was collected from patients at 8:00 am^39^. Sera was separated and stored at − 80 °C before use. Cytokine values (TNF-α, IL-1β, IL-10, IL-12) and glycan—binding molecule Gal-3 are measured by enzyme-linked immunosorbent assay (ELISA) kits (R&D Systems, Minneapolis, Minn, USA). Assay Range: (1) TNF-α: 15.6–1000 pg/mL; (2) IL-1β: 3.9–250 pg/mL; IL-12: 7.8–500 pg/mL; IL-10: 15.6–1000 pg/mL; Gal-3: 600–20,000 pg/mL. Arterial blood gases (oxygen saturation-Sa0_2_, partial pressure of oxygen-p0_2_, partial pressure of carbon dioxide-pC0_2_), of all patients were measured several times during the day by ion selective electrode on the automaton.

All patients had a CXR using digital portable anteroposterior (AP) technique. Radiographic outcomes were described according to Yoon et al.^40^ following 5-point scoring scale: (1) normal; (2) patchy atelectasis and/or hyperinflation and/or bronchial wall thickening; (3) focal consolidation; (4) multifocal consolidation; and 5-diffuse alveolar changes.

We also examined the length of hospital treatment of patients in all stages of the disease, shown through the days.

### Assessment of the functional phenotype of immunocompetent cells in peripheral blood

We used fluorochrome- labeled mAbs: PerCP anti-human CD3 (Miltenyl Biotec), FITC anti-human CD11b (Miltenyl Biotec), PerCP anti-human CD11c (BioLegend), FITC anti-human CD56 (Beckman Coulter), PE anti-human CCR5 (BioLegend) or isotype-matched controls (BD Pharmingen). For intracellular staining, cells stimulated with phorbol 12-myristate 13-acetate (50 ng/mL, Sigma-Aldrich), ionomycin (500 ng/mL, Sigma-Aldrich) and GolgyStop (BD Pharmingen, NJ) for 4 h and stained fluorochrome-labeled anti-human mAb: APC anti-human Gal-3 (BioLegend), PE anti-human IL-1β (BD Pharmingen), PE anti-human TNFα (eBiosciences).

### Statistical analysis

Statistical Analysis Software IBM SPSS (version 23. 0) was used for all data analyses. Kruskal–Wallis test, Chi-square test, and Mann–Whitney *U* test were used. Data were shown as median or mean ± standard error of mean. Pearson’s or Spearman’s correlation assessed the possible relationship between variables. Strength of correlation was defined as negative or positive weak (− 0.3 to − 0.1 or 0.1 to 0.3), moderate (− 0.5 to − 0.3 or 0.3 to 0.5), or strong (− 1.0 to − 0.5 or 0.5 to 1.0). Univariate and multivariate binary logistic regression was performed to investigate association of independently variables (demographics and initial laboratory characteristics) with critical stage of COVID-19. Statistical significance was set at p < 0.05.

## Data Availability

The datasets used and/or analysed during the current study available from the corresponding author on reasonable request.
